# King Edward's Hospital Fund for London

**Published:** 1908-01-11

**Authors:** 


### KING EDWARD'S HOSPITAL FUND FOR LONDON.

A MEETING of the General Council of King Edward's Hospital Fund for London was held on the 3rd inst. at the offices of the Fund, 7 Walbrook, E.C. There were present: Mr. Hugh Colin Smith (in the chair), the president of the Local Government Board (the Right Hon. John Burns, M.P.), the President of the Hospital Sunday and Hospital Saturday Funds (the Lord Mayor), the Governor of the Bank of England (Mr. W. Middleton Campbell), the Right Hon. Sir Savile Crossley, Bart., the Right Hon. Sir Joseph Dimsdale, Bart., the Right Hon. C. Stuart-Wortley, K.C., M.P., Sir W. S. Church, Bart., Lieut.-Colonel Sir Arthur Bigge, Sir Horace Marshall, Sir W. J. Collins, M.P., Sir John Craggs, Dr. Edwin Freshfield, Mr. Frederick M. Fry, Mr. J. G. Griffiths, Mr. J. Danvers Power.

A communication was received signed by his Royal Highness the President appointing for the year 1908 the members of the General Council, the Finance Committee, the Distribution Committee, the Convalescent Homes Committee, and the Executive Committee, and the officers—namely, Hon. Treasurer, Lord Rothschild; Hon. Secretaries, Sir Savile Crossley, Bart., and Mr. Frederick M. Fry; Hon. Solicitors, Messrs. Freshfields; Hon. Auditors, Messrs. Deloittee, Plender, Griffiths and Co.; Secretary, Mr. Maynard.

The resolutions providing for the work of the Fund for 1908, which were approved at the meeting of the President and General Council held at Marlborough House on December 16, 1907, were formally adopted.

